# Metabolomic and cultivation insights into the tolerance of the spacecraft-associated *Acinetobacter* toward Kleenol 30, a cleanroom floor detergent

**DOI:** 10.3389/fmicb.2023.1090740

**Published:** 2023-03-06

**Authors:** Rakesh Mogul, Daniel R. Miller, Brian Ramos, Sidharth J. Lalla

**Affiliations:** ^1^Chemistry and Biochemistry Department, California State Polytechnic University, Pomona, CA, United States; ^2^Blue Marble Institute of Science, Seattle, WA, United States

**Keywords:** *Acinetobacter*, detergent, survival, metabolomics, planetary protection, spacecraft, cleanrooms

## Abstract

**Introduction:**

Stringent cleaning procedures during spacecraft assembly are critical to maintaining the integrity of life-detection missions. To ensure cleanliness, NASA spacecraft are assembled in cleanroom facilities, where floors are routinely cleansed with Kleenol 30 (K30), an alkaline detergent.

**Methods:**

Through metabolomic and cultivation approaches, we show that cultures of spacecraft-associated Acinetobacter tolerate up to 1% v/v K30 and are fully inhibited at ≥2%; in comparison, NASA cleanrooms are cleansed with ~0.8-1.6% K30.

**Results:**

For A. johnsonii 2P08AA (isolated from a cleanroom floor), cultivations with 0.1% v/v K30 yield (1) no changes in cell density at late-log phase, (2) modest decreases in growth rate (~17%), (3) negligible lag phase times, (4) limited changes in the intracellular metabolome, and (5) increases in extracellular sugar acids, monosaccharides, organic acids, and fatty acids. For A. radioresistens 50v1 (isolated from a spacecraft surface), cultivations yield (1) ~50% survivals, (2) no changes in growth rate, (3) ~70% decreases in the lag phase time, (4) differential changes in intracellular amino acids, compatible solutes, nucleotide-related metabolites, dicarboxylic acids, and saturated fatty acids, and (5) substantial yet differential impacts to extracellular sugar acids, monosaccharides, and organic acids.

**Discussion:**

These combined results suggest that (1) K30 manifests strain-dependent impacts on the intracellular metabolomes, cultivation kinetics, and survivals, (2) K30 influences extracellular trace element acquisition in both strains, and (3) K30 is better tolerated by the floor-associated strain. Hence, this work lends support towards the hypothesis that repeated cleansing during spacecraft assembly serve as selective pressures that promote tolerances towards the cleaning conditions.

## 1. Introduction

Maintaining low biological contamination in cleanroom-type facilities are critical components to spacecraft assembly (Rummel, 1992; NASA, 2011; Frick et al., 2014), crewed spacecraft exploration (Spry et al., 2020), delivery of healthcare facilities (Shams et al., 2016; Rutala and Weber, 2019), and the manufacturing of pharmaceuticals (Sandle, 2015). Common to these confined environments and endeavors are the routine cleansing of non-critical hard surfaces and support equipment with ≥70% v/v ethyl alcohol, 70% v/v isopropyl alcohol, 3% v/v hydrogen peroxide, and/or disposable disinfectant wipes containing quaternary ammonium compounds (Agalloco and Carleton, 2007; Rutala et al., 2008; Wong et al., 2013; Frick et al., 2014; Checinska Sielaff et al., 2019).

For pharmaceutical facilities, and to lesser extent healthcare facilities, the floors are additionally cleansed with disinfectants and/or detergents such as benzalkonium chloride (and other quaternary ammonium compounds), hydrogen peroxide, sodium hypochlorite, and other alternatives (Murtough et al., 2001; Rutala et al., 2008; Sandle, 2012; Eissa et al., 2014; Han et al., 2021; Tembo et al., 2022). While debated in efficacy (Daschner et al., 1982; Meyer and Cookson, 2010; Suleyman et al., 2018), the surface cleaning agents are often rotated in these facilities to minimize potential microbial resistance and/or selection toward the cleaning conditions (Murtough et al., 2001; Sandle, 2012).

For NASA cleanrooms (ISO class 8) where Mars and Europa spacecraft are assembled, the floors are routinely cleansed with ~0.8-1.6% v/v Kleenol 30 (Mogul et al., 2018; Danko et al., 2021), which is an alkaline detergent formulation. Due to the proprietary nature of Kleenol 30, scant information is available regarding the precise chemical composition. Available safety data sheets indicate a composition containing sodium dodecyl benzene sulfonate (1% w/w), polyethylene glycol mono-nonylphenyl ether (1–5% w/w), sodium metasilicate (1–5% w/w), ethylenediaminetetraacetic acid (EDTA; 2% w/w), sodium metasilicate (1–5% w/w), potassium hydroxide (KOH; ~2% v/v; presumably prepared from concentrated KOH), and 2-butoxyethanol (10–15% w/w). When considering the mechanism of cleansing for Kleenol 30, the combined chemical ingredients imply adjustments toward alkalinity (KOH), sequestering of transition and alkaline earth metals through chelation by the metal-binding reagents (EDTA and sodium metasilicate), and sequestering, emulsification, and removal of organics (non-polar, polar, and charged organics) by detergent action (polyethylene glycol mono-nonylphenyl ether, dodecyl benzene sulfonate, and sodium metasilicate).

Yet, despite these robust cleaning practices, spacecraft assembly facilities harbor a persistent yet low abundance microbial bioburden (Danko et al., 2021; Hendrickson et al., 2021). Further, molecular measures reveal the presence of a core microbiome across the spacecraft assembly facilities, International Space Station (Checinska et al., 2015; Checinska Sielaff et al., 2019), and clinical and operating facilities (Shams et al., 2016; Ellingson et al., 2020; Perry-Dow et al., 2022). In spacecraft assembly facilities, the microbiomes are reasonably diverse though generally low in total counts with measures of ~10^3^–10^4^ 16S rRNA m^−2^ (intact cells), ~10^4^–10^5^ 16S rRNA m^−2^ (total cells), 1–40 OTU m^−2^, ~10^2^–10^4^ colony forming units m^−2^, and ~ 10^1^ spores m^−2^ (La Duc et al., 2012; Mahnert et al., 2015; Moissl-Eichinger et al., 2015).

Among the dominant members of the core microbiome across spacecraft and healthcare facilities are the *Acinetobacter* (La Duc et al., 2012; Mora et al., 2016; Hendrickson et al., 2021). The *Acinetobacter* are a Gram-negative bacterial genus associated with desiccation tolerance (McCoy et al., 2012; Farrow et al., 2018), radiation tolerance (La Duc et al., 2003; McCoy et al., 2012), bioemulsification (Pirog et al., 2021), biofilm formation (Yeom et al., 2013; Pompilio et al., 2021), and multi-drug resistance (Peleg et al., 2008).

In Mogul et al. (2018), we showed that *Acinetobacter radioresistens* 50v1, which was isolated from the surface of the pre-flight Mars Odyssey orbiter, could be cultivated in the presence of 1% v/v Kleenol 30, a floor detergent, while utilizing ethanol, a surface cleaner, as a sole carbon source. Additionally, cultivations of *A. radioresistens* 50v1 with Kleenol 30 were shown to yield tri, penta, and octaethylene glycols, which is suggestive of partial biodegradation of polyethylene glycol mono-nonylphenyl ether (a component of the Kleenol 30 formulation) *via* scission of the ether linkage (White, 1993) – to yield the mixed polyethylene glycols (e.g., tri, penta, and octaethylene glycol).

In addition, studies show that clinical strains of *Acinetobacter baumanii* tolerate benzalkonium chloride, which is a biocide and surface cleaner, through biofilm formation (Rajamohan et al., 2009) and increased expression of efflux pump genes (Fernández-Cuenca et al., 2015; Srinivasan et al., 2015). Further, survivability studies on cleanroom-associated *Acinetobacter* reveal extreme tolerances toward aqueous hydrogen peroxide, a surface and floor disinfectant; where the tolerances are perhaps the highest among Gram-negative bacteria (McCoy et al., 2012; Derecho et al., 2014; Mogul et al., 2018). These combined survival characteristics are likely key contributors to the *Acinetobacter* being among the dominant members of the spacecraft and healthcare microbiomes.

Therefore, to obtain molecular and quantitative insights into the tolerances of the cleanroom-associated *Acinetobacter*, we measured the impacts of Kleenol 30 on the survivals, cultivation kinetics, and intracellular and extracellular metabolomes. By design, we profiled *Acinetobacter* strains isolated from differing sub-locations within the NASA cleanrooms for spacecraft assembly (e.g., floor and spacecraft surface) which were subjected to differing cleaning regimes (e.g., Kleenol 30 for the floors vs. isopropyl alcohol and/or ethyl alcohol for spacecraft surfaces). Hence, comparisons across these sub-environments lend support toward the hypothesis that repeated cleansing under the conditions of spacecraft assembly serve as selective pressures that favor or promote biochemical tolerances toward the local cleaning conditions.

## 2. Materials and methods

### 2.1. Materials and conditions

Bacterial strains of *A. johnsonii* 2P08AA and *A. radioresistens* 50v1 were obtained through the Planetary Protection Culture Collection at the Jet Propulsion Laboratory. Stocks of ethanol (ENG Scientific) and Kleenol 30 (Mission Laboratories, Los Angeles, CA; Clovis Janitorial) were sterile filtered and stored as 200 μL aliquots at 4°C. Stock solutions of 25 mM Fe^2+^ were prepared using ferrous ammonium sulfate (Fe(NH_4_)_2_(SO_4_)_2_·6H_2_O; EM Science) in ultrapure water (18 MΩ^−1^), followed by sterile filtration (0.2 μm syringe filter, VWR), and storage as 200 μL aliquots at 4°C (with no visible precipitation over long term storage).

Concentrated minimal media (5x MM) were prepared using 30.0 g Na_2_HPO_4_·7H_2_O (Sigma), 15.0 g KH_2_PO_4_ (EM Science), 2.50 g NaCl (Fisher Scientific), 5.00 g NH_4_Cl (EM Science) per liter of ultrapure water. Low-osmolarity media (0.2x M9) were prepared by adding 0.4929 g MgSO_4_·7H_2_O (EM Science) and 0.0147 g CaCl_2_·2H_2_O (EM Science) to 200.0 mL 5x MM and dilution to 1.00 L using ultrapure water (to yield 1x M9), followed by an additional 5-fold dilution (200 mL 1x M9 in 1.00 L) to yield 0.2x M9. Final concentrations for ions in 0.2x M9 were 20 μM Ca^2+^, 400 μM Mg^2+^, 4.4 mM K^+^, 10.7 mM Na^+^, and 3.7 mM NH_4_^+^, along with 5.4 mM Cl^−^, 4.5 mM H_2_PO_4_^−^, 4.4 mM HPO_4_^2−^, and 0.4 mM SO_4_^2−^.

Lysogeny broth media (LB) were prepared using 10.0 g tryptone (VWR), 5.0 g yeast extract (Amresco), 5.0 g NaCl (Fisher Scientific) and 1 mL of 1 M NaOH (Sigma-Aldrich) per liter of ultrapure water. Agar plates were prepared using 1.0 L LB and 15 g agar (Amresco).

Cultivations were performed in 15 mL screw cap test tubes (Pyrex, borosilicate glass; 13 × 100 mm), which were washed with tap water, rinsed with distilled water, and autoclaved between experiments. All screw cap test tubes were capped tightly and wrapped with parafilm during cultivation to prevent loss of ethanol, which served as a sole supplied carbon source. Cultivations were performed with mild agitation (200 rpm) using a New Brunswick Scientific Innova 4,200 incubator. Cell densities were monitored during cultivation by following agar plate counts and changes in optical density (OD) at 600 nm (Spectronic 20 Genesys). All media were autoclaved at 121°C and 15 psi for 45 min.

### 2.2. Cultivations with Kleenol 30

Glycerol stocks of *A. radioresistens* 50v1 and *A. johnsonii* 2P08AA were separately streaked onto LB agar plates and incubated at 32°C for ~24 h. Isolated colonies were inoculated into 2.0 mL 0.2x M9 containing 25 μM Fe^2+^ and 0–2.0% *v/v* Kleenol 30 (or 0–40.0 μL). Pre-cultures were initiated by addition of 20.0 μL 90% *v/v* ethanol to yield final concentrations of 150 mM or 1.0% *v/v* ethanol. Pre-cultures were grown to late log phase (OD ~0.6 at ~12 h). Target cultures were prepared using fresh media (2.00 mL) which were inoculated with 20.0 μL (1:100 dilution) of the respective pre-cultures and initiated by addition of 1.0% *v/v* ethanol.

Temporal changes in OD were followed for 0–15 h. Growth kinetics were characterized by regression analysis (Microsoft Excel) using a modified version of the Gompertz equation (Begot et al., 1996), which describes a non-linear bacterial growth model, and yields the parameters of growth rate (*k*), lag time (*L*), and an estimate of the maximum change in relative biomass ( $\log\left(N/N_{0}\right)$ ). All regressions were minimized by least squares analysis. Control cultures containing no inoculate showed no growth (OD ≤ 0.002), as did cultures containing inoculate but no ethanol (OD ≤ 0.002), which indicated negligible biological contamination and accumulation of abiotic particles during cultivation.

Survivals of late-log phase cultures (OD ~0.4) were assessed by plating onto LB agar plates. In control experiments, plate counts using 0.2x M9 agar plates supplemented with ethanol (just prior to use) yielded irreproducible results, when compared to LB agar plate, likely due to variances in adsorption of ethanol (under our conditions). For the plate count assays, therefore, aliquots (100 μL) of the cultures (in 0.2x M9/Fe) were transferred to 2.5 mL microcentrifuge tubes, decimally diluted by 10^6^-fold using 0.2x M9, and spread (20 μL) onto LB agar plates using sterile plastic cell spreaders. All plates were sealed with parafilm and incubated at 32°C for 24 h. Plates with ≤300 colonies were enumerated and expressed as colony forming using per mL of the parent culture (cfu mL^−1^).

### 2.3. Metabolomics of *Acinetobacter* cultivations in Kleenol 30

Cultures of *A. radioresistens* 50v1 and *A. johnsonii* 2P08AA were prepared as described and harvested during early stationary phase (OD ~0.6–0.7, 7–8 h) by centrifugation (3,500 rpm) at 4°C for 15 min (Beckman Coulter Allegra 21R). After centrifugation, the cell pellets and supernatant fractions were separated and, respectively, treated. The supernatants were saved as 500 μL aliquots, dried using a DNA 110 Savant DNA SpeedVac, and stored at −80°C. The pelleted cells were washed twice with 0.2x M9 and stored at −80°C.

Samples were characterized by the West Coast Metabolomics Center using gas chromatography and time-of-flight mass spectrometry (GC-TOF/MS). In brief, dried cells and culture broth were separately resuspended in 2 mL of pre-chilled (−20°C) and degassed extraction solvent (acetonitrile:isopropanol:water, 3:3:2), vortexed for 30 s, shaken for 5 min at 4°C, clarified by centrifugation (~12,000 g), and the resulting supernatant evaporated to dryness. Samples were derivatized by resuspension in 10 μL of 40 mg/mL methoxyamine hydrochloride in pyridine (30°C, 1.5 h) followed by addition of 41 μL N-methyl-N-(trimethylsilyl) trifluoroacetamide (80°C, 30 min); fatty acid methyl esters (e.g., C8-C30) were additionally added to serve as retention index markers (Barupal et al., 2019).

Samples were transferred to crimp top vials and separated on an Agilent 6,890 Gas Chromatograph equipped with a Gerstel automatic liner exchange system (ALEX), multipurpose sample (MPS2) dual rail, Gerstel CIS cold injection system (Gerstel, Muehlheim, Germany), and built-in gas purifier (Airgas, Radnor PA). Chromatographic separation was afforded using a 30 m (0.25 mm i.d.) Rtx-5Sil MS column (0.25 μm 95% dimethyl 5% diphenyl polysiloxane film) with an additional 10 m integrated guard column (Restek, Bellefonte PA). Carrier gas was 99.9999% pure Helium with a constant flow of 1 mL/min.

Sample volumes of 0.5 μL were injected with a 10 μL s^−1^ injection speed on a spitless injector with a purge time of 25 s. Temperature profile included 1 min at 50°C for the oven temperature, an increase of 20°C min^−1^ to 330°C across 14 min, with a final hold for 5 min at 330°C. A temperature of 280°C was used for the transfer line between the gas chromatograph and Leco Pegasus IV time of flight mass spectrometer (single mass analyzer). The measured mass range was 85–500 Da, scan rate was at 17 spectra s^−1^, electron energy was 70 eV, ion source temperature was 250°C, and detector voltage was 1850 V. Spectra were acquired using the Leco ChromaTOF software vs. 2.32 (St. Joseph, MI) and processed using the BinBase database system (Fiehn et al., 2005, 2010; Trigg et al., 2019), which quantifies (signal-to-noise ratio of 5:1) and matches mass peaks to the Fiehn mass spectral library using retention index (RI) and reference mass spectral information (which are internally compiled by the West Coast Metabolomic Center). Metabolomic data (Study ID ST002380, DatatrackID: 3552) were stored at the NIH Common Fund’s National Metabolomics Data Repository (Sud et al., 2016).

### 2.4. Statistical analyses

Comparisons of the cultivation kinetic parameters were conducted using unpaired Student’s t-tests and one- and two-way ANOVA analyses (Microsoft Excel), where statistical relevance was accepted at *p <* 0.05, normal distribution of the data was supported by Shapiro–Wilk tests (Past 4.10; Hammer et al., 2001), and variances were assumed as equal. Metabolomic data were compared using univariate, multivariate, and visual approaches.

Discrete changes in the metabolomes (e.g., trends for a single metabolite) were identified using unpaired Student’s *t*-tests (Microsoft Excel) with corrections for multiple testing using a false discovery rate (FDR) of FDR ≤ 0.20 (Benjamini and Hochberg, 1995). For the 700 metabolites in the total study (whole cell and extracellular metabolomes from 2 bacterial strains treated with and without Kleenol 30), normal distributions (Shapiro–Wilk Tests) were indicated for ~92% (646) of the metabolites. To account for potential underestimations of normality given the sample size (*n* = 3), metabolites exhibiting thresholds for normality of *p* > 0.03 (Shapiro–Wilk Tests) *and* significance of *p <* 0.05 (Student’s *t*-tests) were carried forward in the univariate and visual assessments of the data.

Broad structural and metabolic trends were obtained by visualizing the changes in the metabolomes (*p <* 0.05) using MetaMapp^1^ and Cytoscape 3.9.1^2^ (Barupal et al., 2012), which constitute a statistical organizational approach to yield visual maps of metabolites arranged by known structural patterns and metabolic pathways. Confirmation of broad changes in the metabolomes were obtained using ChemRich^3^ (Barupal and Fiehn, 2017), a statistical enrichment tool that compares groups of metabolites based on structural and biochemical classes. For this study, the standard and user-defined classifications in ChemRich included amino acids, monosaccharides, sugar acids, organic acids, fatty acids, lipid-related metabolites, nucleotide-related metabolites, and compatible solutes. Multivariate tests were conducted using canonical correspondence analyses (Past 4.10; Hammer et al., 2001) to correlate the metabolomic trends to differing cultivation growth parameters and conditions.

## 3. Results

### 3.1. Cultivation and survival in the presence of Kleenol 30

**Figure 1:**
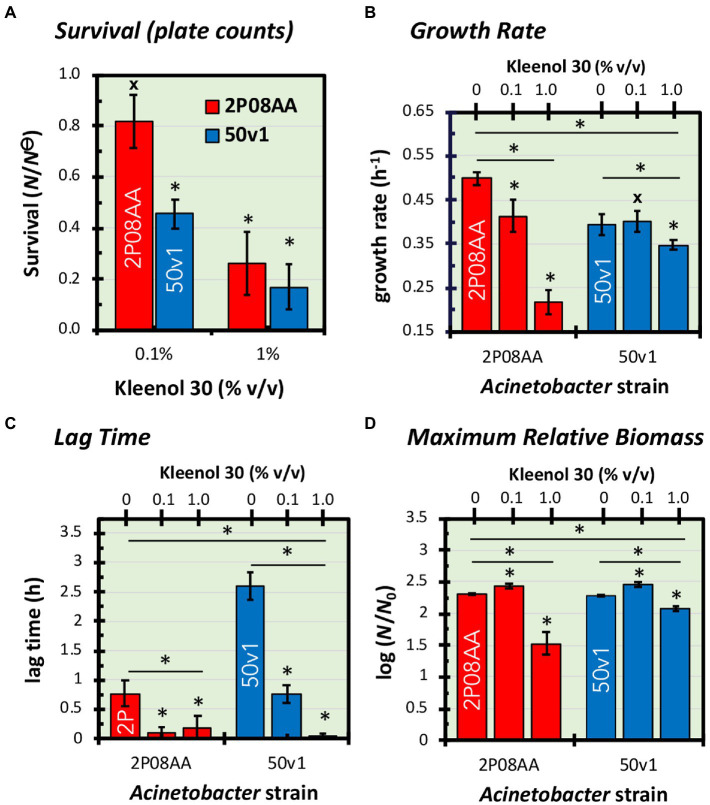
Impacts of 0–1.0% v/v Kleenol 30 (K30) on **(A)** the survival and **(B–D)** cultivation kinetics of *Acinetobacter johnsonii* 2P08AA (red) and *Acinetobacter radioresistens* 50v1 (blue). Survival (*N*/*N*^⊝^) is expressed as the ratio of plate counts (cfu mL^−1^) from cultures grown in the absence of K30 (*N*^⊝^) and presence of K30 **(*N*)**. Fitted regressions of growth curves yielded changes in **(B)** growth rates (*h*^−1^), **(C)** lag time (*h*), and **(D)** maximum relative biomass (log (*N*/*N*_0_)), which is assumed to represent a ratio of the total biomass (*N*) at stationary phase and the biomass at the start of the culture (*N*_0_). Error bars represent the standard errors (*n* = 4–5, growth curves; *n* = 3–5, plate counts). Univariate tests are represented as asterisks (*) for t-tests with *p <* 0.05, hashtags (#) for t-tests with *p* ≥ 0.05, and asterisks (*) with an underlying line for one or two-way ANOVA with *p <* 0.05.

In Figure 1A we show that the cleanroom-associated *Acinetobacter* tested in this study, *A. johnsonii* 2P08AA and *A. radioresistens* 50v1, readily grow in the presence of ≤1% v/v Kleenol 30 (K30) under low-osmolarity aqueous conditions with ethanol serving as the sole supplied carbon source. Cultivations on 2% v/v K30 did not yield measurable cell densities.

In 0.1% v/v K30, cultures of the floor-associated *A. johnsonii* 2P08AA show no apparent loss in survival, as plate counts (~late log phase) effectively show no difference (*p* > 0.05) in the presence (2.1 ± 0.5 × 10^7^ cfu mL^−1^) and absence (2.6 ± 0.1 × 10^7^ cfu mL^−1^) of the detergent. These trends indicate quantitative survival of the 2P08AA strain in the presence of 0.1% v/v K30.

For the spacecraft surface-associated *A. radioresistens* 50v1, in contrast, survivability in 0.1% v/v K30 readily decreases (*p* = 0.006) to 46 ± 6% (propagated error) – as calculated by comparison of cultures cultivated in the presence (4.0 ± 0.4 × 10^7^ cfu mL^−1^) and absence (8.8 ± 0.7 × 10^7^ cfu mL^−1^) of 0.1% v/v K30. These trends are indicative of ~50% survival in the presence of 0.1% v/v K30.

In 1.0% v/v K30, survivabilities for the 2P08AA strain (26 ± 12%) and 50v1 strain (22 ± 8%) decrease to similar values – as indicated by comparison of cultures cultivated in the presence (2P08AA, 0.7 ± 0.3 × 10^7^ cfu mL^−1^; 50v1, 1.9 ± 1.1 × 10^7^ cfu mL^−1^) and absence (2P08AA, 2.6 ± 0.1 × 10^7^ cfu mL^−1^; 50v1, 8.8 ± 0.7 × 10^7^ cfu mL^−1^) of 1.0% v/v K30. These trends are indicative of ~70–80% inhibition in the presence of 1.0% v/v K30 across both strains.

### 3.2. Impacts of Kleenol 30 on the cultivation kinetics

**Figure 2:**
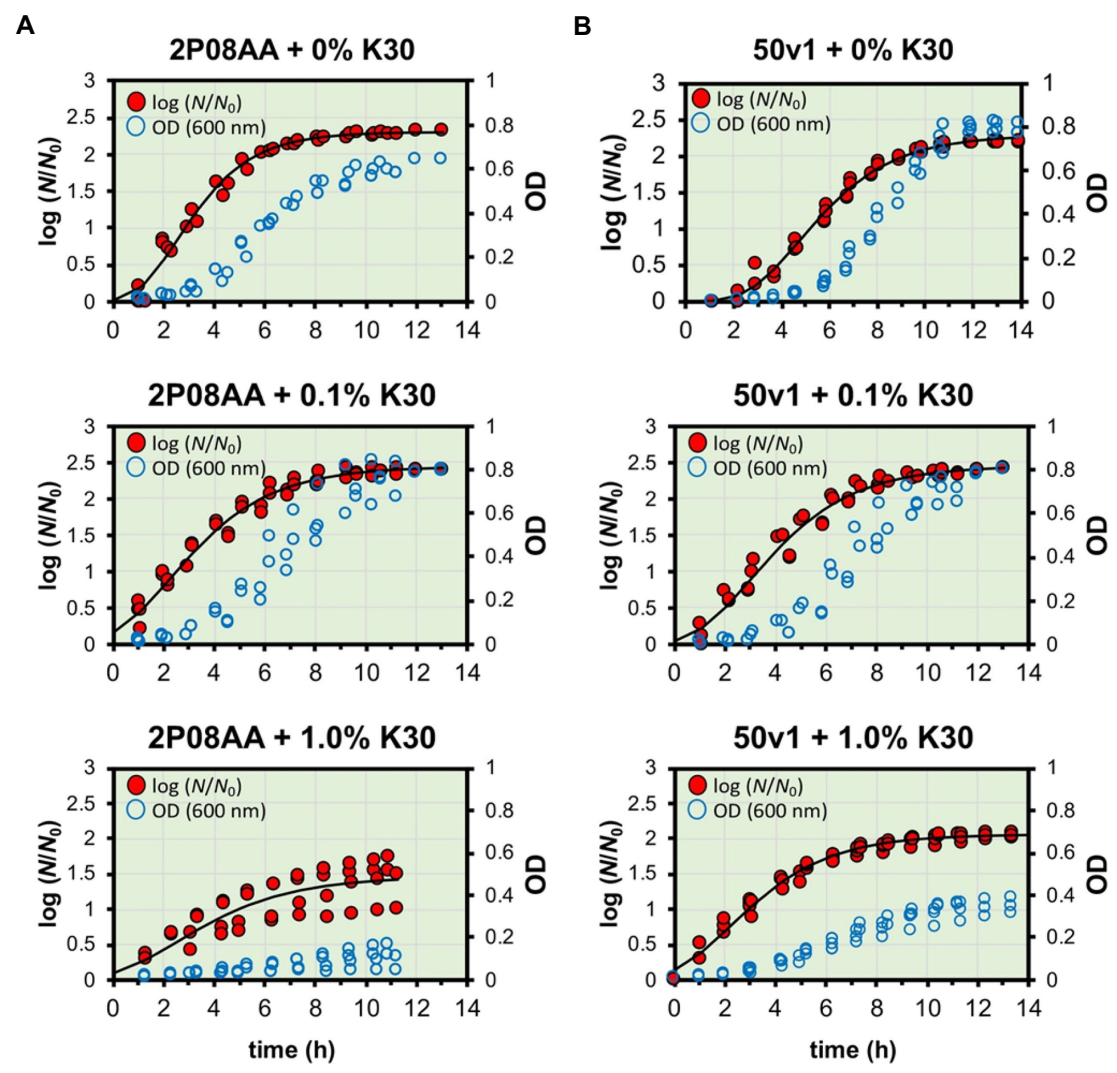
Measured and fitted growth curves for *A. johnsonii* 2P08AA (left panel) and *A. radioresistens* 50v1 (right panel) cultured in the presence of 0–1.0% v/v Kleenol 30 at 28°C in 0.2x M9 media containing 25 μM Fe^2+^ and 150 mM ethanol (the sole supplied carbon source). Optical density measurements (blue empty circles; right-hand y-axis) were converted to log(*N*/*N*_0_) (red filled circles; left-hand y-axis) as described and fit by non-linear regression to yield the parameters of time in lag phase, growth rates, and maximum ratiometric and logarithmic change in biomass (log (*N*/*N*_0_)) at stationary phase.

Displayed in Figures 1B–D and Figure 2 are the cultivation kinetic parameters and associated growth curves for *A. johnsonii* 2P08AA and *A. radioresistens* 50v1. Optical measurements (Figure 2, empty blue circles) for cultures were converted to the ratiometric changes in relative biomass ( $N/N_{0}$ ) by assuming a direct relationship between optical transmittance and cell density for the initial culture media ( $N_{0}$ ) and at the time of measurement ( N ), as outlined in Begot et al. (1996). Changes in relative biomass (Figure 2, filled red circles, fitted line) were expressed as a log function ( $\log\left(N/N_{0}\right)$ ), plotted over time, and fit to a modified version of the Gompertz equation (Eq. 1), as detailed in Begot et al. (1996) and Mogul et al. (2018). Minimized regressions yielded the parameters of growth rate (*k*; cell divisions h^−1^), estimated time in lag phase (*L*; h), and log of the maximum increase in relative biomass ( $\log{\left(N/N_{0}\right)}_{\max}$ ; unitless dimension).

To account for optical scattering, the maximum increases in relative biomass ( $\log{\left(N/N_{0}\right)}_{\max}$ ) were broadly interpreted as maximum increases in intact cells, cellular aggregation, and/or intra- and extracellular polymeric substances at stationary phase. Reported in this study are the averaged values from the minimized regressions along with standard errors (*n* = 3–5 biological replicates).


(1) $$\log\left(\frac{N}{N_{0}}\right)=\log\left(\frac{\Delta OD}{\Delta OD_{min}}\right)=A\cdot \exp\left(-\exp\left(\frac{k\cdot e}{A}\cdot \left(L-t\right)+1\right)\right)$$

Comparisons of the growth rates (*k*) show ~18% decreases for cultivations of the 2P08AA strain in 0.1% v/v K30 (*p =* 0.029) – as calculated by comparison of the difference in growth rates (Figure 1B) between the absence (0.50 ± 0.01 h^−1^) and presence (0.41 ± 0.02 h^−1^) of 0.1% v/v K30. The 50v1 strain, in contrast, shows no change in growth rate (*p =* 0.05) in 0.1% v/v K30 – as calculated by comparison of rates (Figure 1B) in the absence (0.39 ± 0.01 h^−1^) and presence (0.40 ± 0.02 h^−1^) of 0.1% v/v K30. When cultivated in 1.0% v/v K30, growth rates for the 2P08AA strain (0.22 ± 0.03 h^−1^) and 50v1 strain (0.35 ± 0.01 h^−1^) decrease by ~56% (*p =* 0.001) and ~ 13% (*p =* 0.043), respectively, as calculated by comparison rates (Figure 1B) in the absence (2P08AA, 0.50 ± 0.01 h^−1^; and 50v1, 0.39 ± 0.01 h^−1^) of the detergent.

Comparisons by one factor ANOVA analyses confirm that cultivations in the presence of 0.1–1.0% v/v K30 significantly impact the respective growth rates for the 2P08AA (*p =* 0.038, *f* = 4.256) and 50v1 (*p =* 0.0002, *f* = 4.459) strains. Additionally, two factor ANOVA analyses confirm that cultivations in the presence of K30 differentially impact the growth rates of the 2P08AA and 50v1 strains (*p =* 0.015, *f* = 5.318). These combined trends for exponential phase behavior are indicative of higher tolerances toward K30 by the 50v1 strain at 0.1 and 1.0% K30. These trends are opposite to those from plate counts at late-log phase, which indicate a higher tolerance for the 2P08AA strain.

Comparisons of the lag times (*L*) in 0.1 and 1.0% v/v K30 show decreases for both strains during cultivation (Figure 1C), which is unexpected since the addition of detergents was presumed to inhibit growth and increase the time in lag phase. For the 2P08AA strain, the native lag time of 0.77 ± 0.23 h^−1^ (absence of K30) reduces to negligible values (~0 h) in 0.1 and 1.0% v/v K30. For the 50v1 strain, the native lag time of 2.61 ± 0.24 h^−1^ is ~3-fold longer than 2P08AA strain and decreases by ~70% in 0.1% v/v K30 (0.76 ± 0.15 h^−1^) and to negligible values in 1.0% v/v K30.

Comparisons by one factor ANOVA analyses confirm that cultivations in the presence of 0.1–1.0% v/v K30 significantly impact the respective lag times for the 2P08AA (*p =* 0.00008, *f* = 4.256) and 50v1 (*p =* 0.015, *f* = 4.459) strains. Additionally, two factor ANOVA analyses confirm that cultivations in the presence of K30 differentially impact the lag times for the 2P08AA and 50v1 strains (*p =* 0.006, *f* = 5.318). These combined trends indicate that K30 induces accelerated entry into the exponential phase in a concentration dependent manner, where the lag phase for the 2P08AA strain is effectively eliminated at ~10-fold lower K30 abundances when compared to the 50v1 strain – under these conditions.

Comparisons of the maximum change in relative biomass ( $\log{\left(N/N_{0}\right)}_{\max}$ ) for cultivations in 0.1% v/v K30 show unexpected increases for both strains (Figure 1D). For the 2P08AA strain, cultivations in 0.1% v/v K30 yield ~40% increases (*p =* 0.026) in maximum apparent biomass when comparing values obtained in absence (2.30 ± 0.02) and presence of (2.44 ± 0.04) of 0.1% v/v K30 and accounting for the log transformation. Similarly, for the 50v1 strain, cultivations in 0.1% v/v K30 yield ~55% increases (*p =* 0.048) in maximum apparent biomass when comparing values obtained in absence (2.28 ± 0.01) and presence of (2.47 ± 0.04) of 0.1% v/v K30 and accounting for the log transformation. When cultivated in 1.0% v/v K30, the 2P08AA strain (1.53 ± 0.18) and 50v1 strain (2.07 ± 0.03) exhibit decreases of ~80% and ~ 40% in maximum apparent biomass, respectively, which is consistent with the decreased growth rates under these conditions (~56 and ~ 13%, respectively).

Comparisons by one factor ANOVA analyses confirm that cultivations in the presence of 0.1–1.0% v/v K30 significantly impact the respective maximum changes in relative biomass for the 2P08AA (*p =* 0.000003, *f* = 4.256) and 50v1 strains (*p =* 0.000004, *f* = 4.459). Additionally, two factor ANOVA analyses confirm that cultivations in the presence of K30 differentially impact the maximum changes in relative biomass for the 2P08AA and 50v1 strains (*p =* 0.007, *f* = 5.318). Combined, these trends suggest that 0.1% v/v K30 induces changes in apparent biomass in stationary phase.

### 3.3. Impacts of Kleenol 30 on the intracellular metabolomes

#### 3.3.1. Metabolomic considerations

Metabolomes from whole cells of *A. johnsonii* 2P08AA and *A. radioresistens* 50v1 were compared after cultivation in the absence and presence of 0.1% v/v Kleenol 30 (early stationary phase). Per strain, whole cell extracts yielded a total of 119 intracellular metabolites and free metabolites from the membrane (e.g., free fatty acids and monoacylglycerols). Metabolites from both strains showing important changes (*p <* 0.05) due to cultivation with K30 are listed in Table 1, as are changes (fold-changes) in the respective abundances; changes retaining significance (*p <* 0.05, FDR ≤ 0.20) after correction for multiple testing are underlined.

**Table 1:** Numbered list of metabolites from whole cell extracts of *Acinetobacter johnsonii* 2P08AA and *Acinetobacter radioresistens* 50v1, where the magnitude of decreases (↓) or increases (↑) in the relative abundances after cultivation in 0.1% v/v Kleenol 30 (*p* < 0.05) are listed in differing columns and presented in heat-map type coloring to visually represent the magnitude of decreasing (↓: light to dark blue) or increasing (↑: light green to dark red) abundances; the column label of ID# represents the metabolites numbered in Figure 3, CID is the associated PubChem Compound ID, RI is the associated retention index, m/z is the mass to charge ratio for the associated derivatized metabolite, fold-changes represent the ratio of abundances obtained in presence and absence of the K30, asterisks (*) indicate relevance after correction for multiple testing (FDR ≤ 0.20), dashes indicate that the metabolite was not detected, and gray boxes indicate that the option is not applicable.

Whole cell metabolites					2P08AA		50v1	
ID#	Biochemical class	CID	RI	*m*/*z*	Fold-change			
Amino acids					↓	↑	↓	↑
1	Glycine	750	368,707	248	–	–	1.7	
2	Lysine	5,962	663,483	156	–	–	4.8*	
3	Proline	145,742	364,523	142	–	–	6.0	
4	Serine	951	395,020	218	–	–	1.7	
5	Valine	6,287	313,502	144	–	–	2.2	
6	β-alanine	239	435,564	248	–	–	2.1	
7	Citrulline	750	621,404	157	–	–	2.9	
8	Homoserine	12,647	396,135	146	–	–	2.4*	
9	Ornithine	6,262	594,051	174	–	–	2.9*	
10	Alanine–alanine	5,484,352	636,898	188	–	–	1.7	
Dicarboxylic acids					**↓**	**↑**	**↓**	**↑**
11	Fumaric acid	444,972	390,016	245	–	–		2.2
12	Malic acid	525	463,180	233	–	–		2.2
13	Tartartic acid	444,305	534,291	292	–	–		1.4
Sugar acids					↓	**↑**	**↓**	**↑**
14	Gluconic acid	6,857,417	693,148	333		5.1	–	–
15	3-phosphoglycerate	724	610,734	227	–	–	4.0	
16	Glyceric acid	439,194	377,495	189	–	–	4.1	
Monosaccharides					**↓**	**↑**	**↓**	**↑**
17	Arabinose	6,902	550,621	217	1.8		1.9	
18	Mannitol	6,251	663,215	319	–	–	3.7*	
19	Sorbitol	5,780	667,922	217		4.9	–	–
Fatty acids and lipids					**↓**	**↑**	**↓**	**↑**
20	Behenic acid (22:0)	8,215	920,648	117		15	–	–
21	Ethanolamine	700	344,719	174		2.5	–	–
22	Isopentadecanoic acid	151,014	663,518	117	–	–	2.1	
23	Phosphoethanolamine	1,015	604,335	299	2.9		–	–
24	Stearic acid	5,281	787,622	117	–	–		1.5
Nucleotide-related					**↓**	**↑**	**↓**	**↑**
25	5’-AMP	6,083	1,038,688	169	–	–	4.5*	
26	5’-CMP	6,131	700,635	243	–	–		1.6
27	5′-MTA	439,176	967,036	236	–	–	4.0*	
28	pyrophosphate	1,023	327,517	110	–	–	2.1	
Pyrimidines					↓	**↑**	**↓**	**↑**
29	Thymidine	5,789	349,402	170	–	–	1.4	
30	Uracil	1,174	385,735	241	–	–	2.1	
Other					**↓**	**↑**	**↓**	**↑**
31	2,5-dihydroxypyrazine	23,368,901	397,526	241	–	–	4.1*	

**Figure 3:**
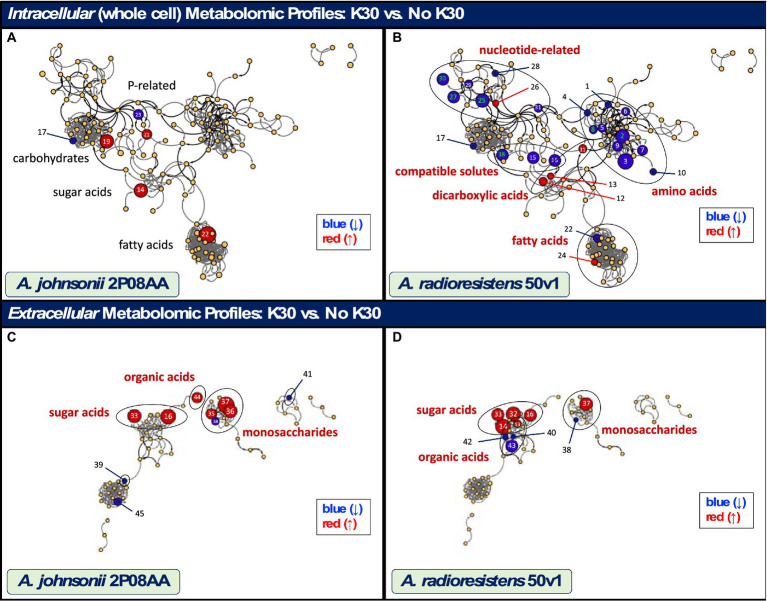
Maps showing the impacts of cultivation in 0.1% v/v K30 on the intracellular and extracellular metabolomes of *A. johnsonii* 2P08AA **(A–C)** and *A. radioresistens* 50v1 **(B,D)**. Metabolites are represented as nodes and organized by structural and metabolic connections (MetaMapp and Cytoscape 3.9.1). Important changes in abundance (*p <* 0.05) are represented as red (increases), blue (decreases), and yellow (no change) nodes, where node sizes represent the degree of change. Relevant metabolites that show important (black text; *p <* 0.05) and significant changes (red text; *p <* 0.05; FDR ≤ 0.20), as listed in Tables 1, 2, are displayed using the associated identity numbers (ID#). Shaded circles represent biochemical classes (red text) that show significant changes (ChemRich); biochemical classes showing no change are listed for comparison purposes (black text).

Displayed in Figure 3 are metabolomic maps for the 2P08AA and 50v1 strains, which readily show the changes resulting from cultivation in K30. The metabolomic maps (MetaMapp graphs) in Figure 3 are organized *via* networks of KEGG reactant pairs (black edges or arrows) and Tanimoto chemical similarity scores (gray edges or arrows). Detected metabolites are presented as nodes (circles). Important changes are highlighted as red nodes (increases in relative abundance), blue nodes (decreases in relative abundance), or yellow nodes (no change). Node sizes signify the relative degree of change (or fold-change).

For the 2P08AA strain (Figure 3A), cultivations in 0.1% v/v K30 yield no statistically discernable impacts to the whole cell metabolome. No changes are observed after accounting for multiple testing (FDR ≤ 0.20, *n* = 119). No changes are observed when parsing the data using ChemRich. While potential increases (*p* < 0.05) are observed in gluconate, analyses in ChemRich provide no support for broad changes in sugar acid content. Despite the potential (*p* < 0.05) decreases in arabinose and increases in sorbitol, analyses in ChemRich show no support for broad changes in monosaccharide composition. Similarly, ChemRich analyses provide no support for broad changes in lipid-related metabolites despite the potential (*p* < 0.05) decreases in phosphoethanolamine and increases in ethanolamine and behenic acid (22:0; long chain saturated fatty acid).

In contrast, the 50v1 strain displays *multiple* changes in the intracellular metabolome due to cultivation in 0.1% v/v K30 (Table 1). After correction for multiple testing (n = 119), significant decreases are observed for lysine, homoserine, ornithine, mannitol, adenosine-5′-monophosphate (5’-AMP), 5′-methylthioadenosine (5′-MTA), and 2,5-dihydroxypyrazine. The whole cell metabolomic maps for the 50v1 strain in Figure 3B were parsed using MetaMapp and ChemRich and expand on the univariate analyses to reveal differential changes in amino acids, compatible solutes, nucleotide-related metabolites, dicarboxylic acids, sugar acids, and saturated fatty acids.

Impacts to amino acids (*p =* 9.1×10^−6^; *Q* = 6.7 × 10^−5^; ChemRich) are supported by decreases in the relative abundances of standard amino acids (glycine, lysine, proline, serine, and valine), non-standard amino acids (β-alanine, homoserine, ornithine, and citrulline), and a peptide (alanine–alanine). Dimerization of glycine under the analytical conditions is suggested by detection of 2,5-dihydroxypyrazine (Haffenden and Yaylayan, 2005) with the observed decreases in 2,5-dihydroxypyrazine in cultures with K30 being generally consistent with the decreases in glycine.

Impacts to compatible solutes (*p =* 9.6×10^−6^; *Q* = 6.7 × 10^−5^; ChemRich) are supported by decreases in mannitol (Empadinhas and da Costa, 2008; Tam et al., 2022), as well as glycerate and 3-phosphoglycerate, which are involved in the biosynthesis of compatible solutes (Franceus et al., 2017). Impacts to nucleotide-related metabolites (*p =* 5.2×10^−4^; *Q* = 2.4 × 10^−3^; ChemRich) are supported by decreases in 5’-AMP, MTA, pyrophosphate, thymidine, and uracil, along with increases in cytidine-5′-monophosphate (5’-CMP). Impacts to dicarboxylic acids (*p =* 4.8×10^−3^; *Q* = 1.7 × 10^−2^; ChemRich) are supported by increases in fumarate, malate, and tartrate. No impacts to saturated fatty acids are observed (*p =* 0.11; *Q* = 0.31; ChemRich) despite the decreases in isopentadecanoic acid (branched saturated fatty acid; 13-methyl-14:0, or 15:0 iso) and increases in stearic acid (saturated fatty; 18:0).

#### 3.3.2. Metabolic considerations

Cross referencing of the metabolomic changes to the KEGG database yields probable impacts to several intracellular pathways for the 50v1 strain. Provided in parentheses are the associated KEGG pathway or reaction identifiers.

Inhibition or reduction in Glycine, Serine, and Threonine Metabolism (map00260) is supported by decreases in intracellular glycerate, 3-phosphoglycerate, serine, glycine, and homoserine. Inhibition or reduction in Lysine Biosynthesis (map00300) and Valine, Leucine, and Isoleucine Biosynthesis (map00290) is supported by decreases in lysine and valine; however, no other changes are noted in the respective pathways.

Inhibition or reduction in proline and arginine biosynthesis through Arginine and Proline Metabolism (map00330) and Arginine Biosynthesis (map00220) are supported by decreases in ornithine, citrulline, and proline. The lack of changes in abundances for urea indicate no measurable impact on the flux of the urea cycle – despite the decreases in ornithine and citrulline. The lack of observed arginine is suggestive of conversion of arginine to ornithine under the analytical conditions (Halket et al., 2005) or the limited presence of free arginine in the cell.

Inhibition or reduction in Peptidoglycan Biosynthesis (map00550) and/or peptide (protein) synthesis is suggested by decreases in alanine–alanine. Inhibition or reduction in β-alanine metabolism (map00410) is supported by decreases in β-alanine, which in turn is suggestive of reductions in arginine and uracil degradation. Differential changes in fatty acid metabolism (map01212) are suggested by the decreases in free isopentadecanoic acid (Reaction R02663) and increases in free stearic acid.

Inhibition or altered flux through glycolysis (map00010) is suggested through decreases in 3-phosphoglycerate. Activation or altered flux through components of the TCA cycle are suggested by increases in fumarate and malate. Inhibition or reduced flux of selected monosaccharides through Fructose and Mannose Metabolism (map00051) and Pentose and Glucuronate Interconversions (map00040) are, respectively, suggested by decreases in mannitol and arabinose.

Differential changes in Nucleotide Metabolism (map01232) are suggested by increases in 5’-CMP and 5′-MTA, along with decreases in 5’-AMP, pyrophosphate, thymidine, and uracil. Additionally, inhibition or reduction in Pyrimidine Metabolism (map00240) is suggested decreases by uracil and thymidine.

### 3.4. Impacts of Kleenol 30 on the extracellular metabolomes

Displayed in Figure 3 are biochemical maps that compare the extracellular metabolomes from *A. johnsonii* 2P08AA and *A. radioresistens* 50v1 (early stationary phase). Per strain, dried cultivation broth (cell free) yielded a total of 56 metabolites, which were processed and visualized as described above. Listed in Table 2 are the extracellular metabolomic changes in the 2P08AA and 50v1 strains after cultivation in the absence and presence 0.1% v/v K30. Pathway analyses were not conducted for extracellular metabolites.

**Table 2:** Numbered list of extracellular metabolites from cultures of *A. johnsonii* 2P08AA and *A. radioresistens* 50v1, where the magnitude of decreases (↓) or increases (↑) in the relative abundances after cultivation in 0.1% v/v Kleenol 30 (*p* < 0.05) are listed in differing columns and presented in heat-map type coloring to visually represent the magnitude of decreases (↓: light to dark blue) or increases (↑: light green to dark red); the ID# represents the metabolite number from Figure 3 and continues with the numbering order from Table 1, CID is the PubChem Compound ID, RI is the associated retention index, m/z is the mass to charge ratio for the associated derivatized metabolite, fold-changes represent the ratio of abundances obtained in presence and absence of the K30, asterisks (*) indicate relevance after correction for multiple testing (FDR ≤ 0.20), dashes indicate that the metabolite was not detected, gray boxes indicate that the option is not applicable.

Extracellular metabolites					2P08AA		50v1	
ID#	Biochemical class	CID	RI	*m*/*z*	Fold-change			
Sugar acids					**↓**	**↑**	**↓**	**↑**
32	Galactonic acid	128,869	690,882	292	–	–		36*
14	Gluconic acid	6,857,417	693,148	333	–	–		46*
33	Gluconolactone	7,027	645,815	220		22		8.8*
16	Glyceric acid	439,194	377,495	189		34		9.3*
34	2-Methylgyceric acid	560,781	372,491	219				1.9
Monosaccharides					**↓**	**↑**	**↓**	**↑**
35	Fructose	439,709	639,442	307		4.5	–	–
36	Galactose	439,357	647,344	319		22	–	–
37	Mannose	18,950	645,856	205		57		20*
38	Ribose	5,779	553,078	217	2.5		1.9*	
Di and monocarboxylic acids					**↓**	**↑**	**↓**	**↑**
39	Adipic acid	196	474,435	111	1.9		–	–
13	Tartaric acid	444,305	534,291	292	–	–		2.9*
40	Citramalic acid	1,081	456,203	247	–	–	1.6	
41	3,4-Dihydroxybenzoic acid	72	620,200	193	2.0		–	–
42	4-Hydroxybutanoic acid	10,413	325,027	233	–	–	2.4*	
43	α-ketoglutarate	51	507,392	198	–	–	9.0*	
44	Oxalic acid	971	260,513	190		5	–	–
Fatty acids					**↓**	**↑**	**↓**	**↑**
45	Pelargonic acid	8,158	399,229	117	3.1		–	–

When grouped by biochemical classes, cultivations of the 2P08AA strain in K30 yield differential impacts to extracellular sugar acids, monosaccharides, and organic acids (di- and monocarboxylic acids). Yet, univariate tests show no changes after correction for multiple testing. Impacts to sugar acids (*p =* 1.4×10^−5^; *Q* = 6.9×10^−5^; ChemRich) are supported by substantial increases in extracellular gluconolactone (22-fold) and glycerate (34-fold). Impacts to di- and monocarboxylic acids, which were grouped as organic acids (*p =* 3.0×10^−4^; *Q* = 7.6×10^−4^; ChemRich), are supported by increases in oxalate and decreases in adipic acid (hexanedioic acid) and 3,4-dihydroxybenzoate (protocatechuate). Impacts to extracellular monosaccharides (*p =* 7.6×10^−3^; *Q* = 1.3×10^−2^; ChemRich) are supported by substantial increases in extracellular fructose (4.5-fold), galactose (22-fold), and mannose (57-fold) and decrease (2.5-fold) in extracellular ribose (pentose).

In contrast, cultivations of the 50v1 strain in K30 – after correction for multiple testing (*n* = 56) – yield significant changes in the extracellular galactonate, gluconate, gluconolactone, glycerate, mannose, ribose, tartrate, 4-hydroxybutyrate, and α-ketoglutarate. Likewise, grouping by biochemical classes are reveals significant changes in the extracellular metabolome.

Impacts to sugar acids (*p =* 1.9×10^−8^; *Q* = 9.6×10^−8^; ChemRich) are supported by substantial increases in extracellular galactonate (36-fold), gluconate (46-fold), gluconolactone (8.8-fold), glycerate (9.3-fold), and 2-methylglycerate (1.9-fold). Impacts to extracellular organic acids (*p =* 7.8×10^−2^; *Q* = 0.16; ChemRich) are suggested by the significant increases in tartrate and significant decreases in α-ketoglutatate, along with the potential decreases in citramalate and 4-hydroxybutyrate. Impacts to extracellular monosaccharides (*p =* 9.6×10^−2^; *Q* = 0.16; ChemRich) are suggested by significant increases in extracellular mannose (20-fold) and significant decreases in ribose (2.5-fold).

### 3.5. Impacts of Kleenol 30 on cultivation and the intracellular metabolome

#### 3.5.1. Canonical correlation analyses

**Figure 4:**
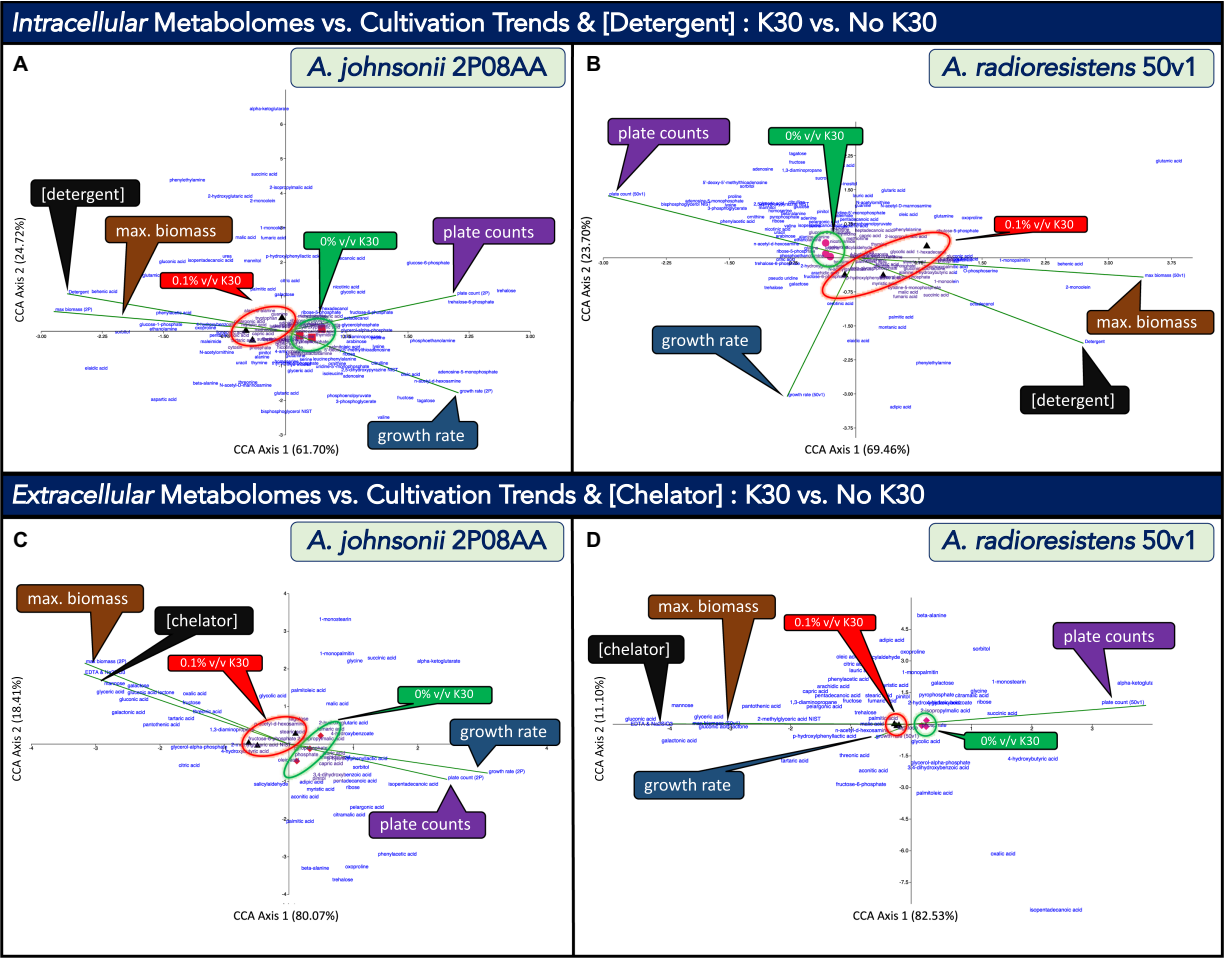
Canonical correspondence analyses (CCA) that compare the impacts of cultivation in 0.1% v/v Kleenol 30 (K30) on **(A)** the intracellular metabolome of *A. johnsonii* 2P08AA, **(B)** the extracellular metabolome of *A. johnsonii* 2P08AA, **(C)** the intracellular metabolome of *A. radioresistens* 50v1, and **(D)** the extracellular metabolome of *A. radioresistens* 50v1 against the quantitative descriptors of growth rates at exponential phase (blue callout boxes, green vectors), plate counts at late-log phase (purple callout boxes, green vectors), maximum relative biomass at stationary phase (brown callout boxes, green vectors), and either the total concentrations of detergents (0.025% w/w; assumed) or chelators (0.02% w/w; assumed) in the cultivation media (black callout boxes, green vectors); dimension reduction of the triplicate metabolomic measures associated with 0% v/v K30 (red squares, pink circles, and red and pink diamonds) and 0.1% v/v K30 (black triangles) are provided, reduced terms are highlighted by the green and red circles and callout boxes, and metabolites are arrayed as text.

In Figure 4, we use canonical correlation analyses (CCA) to, respectively, compare metabolomes from *A. johnsonii* 2P08AA (*n* = 3) and *A. radioresistens* 50v1 (*n* = 3) to differing quantitative descriptors characterizing changes in the cultivation media and growth kinetics in the absence and presence of 0.1% v/v K30. Correlations for the intracellular (or whole cell) metabolomes from late log-phase cultures were assessed against growth rates at exponential phase, plate counts at late-log phase, maximum relative biomass at stationary phase, and total detergent concentrations in the media, which were assumed to be 0.025% w/w in cultures containing 0.1% v/v K30.

The CCA plots include the total array of detected metabolites (text), listed quantitative descriptors (vector lines), and transformed data after dimension reduction (glyphs). For the 2P08AA and 50v1 strains, stronger correlations along CCA Axis 1 (61.70, 69.46%) are observed when compared to CCA Axis 2 (24.72, 23.70%), respectively. Dimension reduction for the metabolomes from the 2P08AA and 50v1 strains (Figures 4A,B) respectively show clear separations between samples prepared in the absence and presence of 0.1% v/v K30. These trends suggest that the intracellular metabolomic compositions of both strains are impacted by K30 during cultivation. Described in the following sub-sections are comparisons to the quantitative descriptors. For selected metabolites, additional clarifications and/or fatty acid abbreviations are provided in parentheses.

#### 3.5.2. Comparisons to survival

When considering changes in survival (Figure 4A), the vector describing plate counts for the 2P08AA strain (late log phase) overlaps with the reduced terms associated with 0% v/v K30 (filled red squares). This correlation suggests that survival trends for the 2P08AA strain (across 0 to 0.1% v/v K30) are associated with limited changes in the intracellular metabolome. This assessment is consistent with the associated univariate tests which indicate no changes in plate counts (*p* > 0.05) or metabolite abundances for the 2P08AA strain in 0.1% v/v K30 (FDR > 0.20).

From the CCA plot for the 2P08AA strain (Figure 4A), metabolites trending alongside the plate counts include trehalose, trehalose-6-phosphate, fructose-6-phosphate, and potentially glucose-6-phosphate. Univariate tests infer no changes in the abundances for these metabolites (*p* > 0.05 or FDR > 0.20). These trends suggest that the quantitative survival for the 2P08AA strain (in part) relates to lack of changes in central metabolism and compatible solute formation.

For the 50v1 strain (Figure 4B), the vector describing plate counts overlaps with the reduced terms associated with 0% v/v K30 (pink circles). This correlation suggests that survival trends for the 50v1 strain (across 0 to 0.1% v/v K30) are associated with limited changes in the intracellular metabolome. This assessment is contrary to the associated univariate tests that support ~50% reductions in survival (*p* < 0.05) and substantial changes in the metabolome (*p* < 0.05, FDR ≤ 0.20).

From the CCA plot for the 50v1 strain (Figure 4B), metabolites trending alongside the plate counts include 4-hydroxybenzoate, several amino acids (similar to those in Table 1), ribose-5-phosphate, N-acetylglucosamine, nicotinic acid, phenylacetic acid, bisphosphoglyercol, and potentially 5’-AMP and 5′-MTA. These trends suggest that survival of the 50v1 strain is related to changes in central metabolism, amino acid metabolism, nucleotide metabolism, and cell wall metabolism (e.g., metabolism of glycosoaminoglycans and peptidoglycans, which are based on N-acetylglucosamine).

Trends from the CCA plot for the 50v1 strain (Figure 4B) are additionally suggestive of changes in native benzene metabolism (e.g., phenylacetic acid and 4-hydroxybenzoate). However, univariate analyses yield no support (*p >* 0.05) for the *apparent* decreases in phenylacetic acid (~0.1-fold decreases) and 4-hydroxybenzoate (~0.9-fold decreases). Alternatively, described further in Section 3.5.3, phenylacetic acid and 4-hydroxybenzoate are potential biodegraded products of K30. Hence, trends for the 50v1 strain hint at a potential for biodegradation of K30 through benzene metabolism.

#### 3.5.3. Comparisons to maximum relative biomass

When considering biomass, the vector describing the maximum relative biomass at stationary phase for the 2P08AA strain (Figure 4A) overlaps with the reduced terms associated with 0.1% v/v K30 (black triangles). This correlation suggests that trends in the maximum relative biomass for the 2P08AA strain (across 0 to 0.1% v/v K30) are associated with changes in the intracellular metabolome. This assessment is consistent with the associated univariate tests for the cultivation data that support increases in the maximum relative biomass (*p* < 0.05), yet counter to the associated univariate tests for the metabolomic data that indicate no change in the relative metabolite abundances *after* correction for multiple testing (FDR > 0.20).

From the CCA plot for the 2P08AA strain (Figure 4A), the metabolites trending alongside the maximum relative biomass include methionine, lignoceric acid (fatty acid; 23:0), oxoproline, and glucose-1-phosphate. Univariate tests indicate no change in the relative abundances for these metabolites (FDR > 0.20). These combined trends suggest that increases in maximum relative biomass relate to minimal or no changes in glucose metabolism, amino acid metabolism, and unsaturated fatty acid abundances.

For the 50v1 strain (Figure 4B), the vector describing the maximum relative biomass overlaps with the reduced terms associated with 0.1% v/v K30 (black triangles). This correlation suggests that trends in the maximum relative biomass for the 50v1 strain (across 0 to 0.1% v/v K30) are associated with changes in the intracellular metabolome. This assessment is consistent with the associated univariate tests on the cultivation data (*p* < 0.05) and metabolomic data (*p* < 0.05, FDR ≤ 0.20), which yield support for increases in the maximum relative biomass and differential changes in metabolite abundances. In contrast to the 2P08AA strain, these trends for 50v1 strain suggest that increases in the maximum relative biomass are associated with several intracellular metabolomic changes.

From the CCA plot for the 50v1 strain (Figure 4B), metabolites trending alongside the increases in maximum relative biomass include lignoceric acid (fatty acid; 23:0), tryptophan, xanthine, stearic acid (fatty acid; 18:0), α-ketoglutarate, phosphate, 4-hydroxybutanoate (4-hydroxybutyric acid), 4-aminobutanoate (4-aminobutyric acid), O-phosphoserine, 1-monopalmitin (1-palmitoylglycerol; palmitoyl = 16:0), behenic acid (fatty acid; 22:0), and 2-monoolein (2-oleoylglycerol; oleoyl = 18:1^Δ9^). These trends suggest that increases in the maximum relative biomass for the 50v1 strain are related (*at the minimum*) to changes in butanoate metabolism (e.g., 4-hydroxybutanoate and 4-aminobutanoate), amino acid metabolism (e.g., tryptophan, α-ketoglutarate, and O-phosphoserine) and phosphate metabolism (phosphate and O-phosphoserine).

The trends for the 50v1 strain (Figure 4B) also suggest a relation to changes in free fatty acids and monoacylglycerols; where the associated metabolites from the CCA plot (and respective univariate analyses in parentheses) include lignoceric acid (~1.2-fold increases; *p >* 0.05), stearic acid (~1.5-fold increases; *p >* 0.05), 1-monopalmitin (~2.2-fold increases; *p >* 0.05), behenic acid (~10-fold increases; *p >* 0.05), and 2-monoolein (~32-fold increases; *p >* 0.05). However, in comparison, ChemRich analyses yield no support for changes in the intracellular fatty acids.

Further, trends from the CCA plot for the 50v1 strain (Figure 4B) support a correlation between the maximum relative biomass and metabolism of polyhydroxyalkanoates (PHAs), which are intracellular polymers (e.g., poly-4-hydroxybutanoate and/or other co-polymers) used for carbon and energy storage (Khanna and Srivastava, 2005). Among the metabolites trending alongside the maximum relative biomass are α-ketoglutarate, glutamate, 4-aminobutanoate, and 4-hydroxybutanoate. These combined metabolites represent a stepwise pathway toward the synthesis of PHAs (KEGG map 00250 and map00650), where oxidized carbons are acquired through the TCA cycle (α-ketoglutarate) and sequentially shuttled through amino acid metabolism (glutamate) and butanoate metabolism (4-aminobutanoate) to yield a monomeric unit (4-hydroxybutanoate) commonly found in PHAs.

Together, these observations are relevant since the accumulation of PHAs can result in increases in relative optical densities (Slaninova et al., 2018), which is consistent with our growth curves and ensuing regression analyses that yield increases in the maximum relative biomass in the presence of 0.1% v/v K30. Hence, for the 50v1 strain, the increases in maximum relative biomass may relate to the increased synthesis of PHAs under the stresses imposed by K30.

#### 3.5.4. Comparisons to growth rates

When considering growth rates (exponential phase), the vector for the 2P08AA strain (Figure 4A) overlaps with the reduced terms associated with 0% v/v K30 (red squares). This correlation suggests that growth rates trends for the 2P08AA strain (across 0 to 0.1% v/v K30) are associated with limited changes in the intracellular metabolome. This assessment is consistent with the associated univariate tests for growth rates that support moderate decreases of ~18% (*p* < 0.05) and the associated univariate tests for the metabolomes that imply no change in relative abundances (FDR > 0.20).

From the CCA plot of the 2P08AA strain, metabolites trending alongside the growth rates include palmitoleic acid (fatty acid; 16:1^Δ9^), 5′-MTA, ribose, several amino acids, oleic acid, N-acetylglucosamine, and 5’-AMP. These trends suggest that the decreases in growth rates are related to changes in amino acid metabolism, cell wall metabolism, and nucleotide metabolism. The trends also suggest relations to changes in free unsaturated fatty acid content through associations with palmitoleic acid (~0.7-fold decrease; *p >* 0.05) and oleic acid (~0.3-fold decrease; *p >* 0.05). However, ChemRich analyses yield no support for changes in unsaturated fatty acid metabolism.

For the 50v1 strain (Figure 4B), in contrast, the vector describing growth rates overlaps more closely with the reduced terms associated with 0.1% v/v K30 (black triangles). This correlation suggests that growth rate trends for the 50v1 strain (across 0 to 0.1% v/v K30) are associated with changes to the intracellular metabolome. In comparison, univariate tests on the cultivation data indicate no change in the growth rates (*p* > 0.05), while univariate tests on the metabolomic data support changes in the abundances for multiple metabolites (*p* < 0.05, FDR ≤ 0.20). These combined trends are suggestive of substantial post-exponential phase changes in the metabolomes, as growth rates for the 50v1 strain during exponential phase are unaltered by – and potentially not associated with – the multiple metabolomic changes observed during late-log phase.

#### 3.5.5. Comparisons to detergent concentrations

When considering the K30 formulation (Figures 4A,B), the vectors describing detergent concentrations (~0–0.025% w/w) overlap with the reduced terms associated with 0.1% v/v K30 for both the 2P08AA and 50v1 strains (black triangles). These correlations suggest the metabolomes from both strains adjust in response to detergents in 0.1% v/v K30. We note the trends for detergent concentrations likely overlap with the maximum relative biomass term, which plots across the same respective quadrant.

For the 2P08AA strain, metabolites trending alongside the detergent concentrations include myristic acid (fatty acid; 14:0), pelargonic acid (fatty acid; 9:0), 4-hydroxybenzoate, phenylacetic acid, and behenic acid (fatty acid; 22:0). The trends for 4-hydroxybenzoate and phenylacetic acid lend support toward the potential for biodegradation of dodecyl benzene sulfonate and polyethylene glycol mono-nonylphenyl ether, the detergents from the K30 formulation. As described in Hashim et al. (1992), phenylacetic acid is a potential product of microbial degradation of dodecyl benzene sulfonate through oxidation (or metabolism) of the dodecyl group (to yield 4-sulfonylphenylacetic acid), followed by desulphonization (to yield phenylacetic acid). Similarly, we posit that biodegradation of polyethylene glycol mono-nonylphenyl ether could yield 4-hydroxybenzoate through scission of the polyethylene glycol units (to yield 4-nonylphenol), as described in Section 1 followed by oxidation or metabolism of the nonyl side group (to yield 4-hydroxybenzoate). In comparison, however, univariate tests yield no support for changes in the abundances for phenylacetic acid (~2.4-fold increases; *p* = 0.39) and 4-hydroxybenzoate (~1.5-fold increases; *p* = 0.11).

The trends for the 2P08AA strain are also suggestive of correlations between detergent concentrations and changes in free fatty acid content. Relevant metabolites from the CCA plot (and the associated univariate analyses in parentheses) include behenic acid (~15-fold increases; *p* < 0.05), myristic acid (~1.2-fold increases; *p* > 0.05), and pelargonic acid (~1.4-fold increases; *p* > 0.05). We note that behenic acid levels in fluvial biofilms increase after exposure to desiccation (Serra-Compte et al., 2018). We also speculate the increases in long chain saturated fatty acid content may promote aggregation with the detergents in the intracellular and/or membrane space, which could potentially serve as a survival mechanism and/or acquisition strategy during biodegradation. Analyses by ChemRich, however, yield no support for changes in fatty acid or lipid-related metabolites.

For the 50v1 strain (Figure 4B), metabolites that trend alongside detergent concentrations include tartrate, 1-monoolein (1-oleoylglycerol; oleoyl = 18:1^Δ9^), and octadecanol (fatty alcohol). These trends are suggestive of increases in an intracellular dicarboxylic acid (tartrate) and free lipid-related molecules (1-monoolein and octadecanol), which may, respectively, relate to metal retention in the cell and the aggregation of detergents in the intracellular or membrane space.

### 3.6. Impacts of Kleenol 30 on the cultivations and extracellular metabolomes

#### 3.6.1. Canonical correlation analyses

In Figure 4, we use canonical correlation analyses (CCA) to, respectively, compare the extracellular metabolomes from *A. johnsonii* 2P08AA (*n* = 3) and *A. radioresistens* 50v1 (*n* = 3) from late log-phase cultures to the quantitative descriptors of growth rates at exponential phase, plate counts at late-log phase, maximum relative biomass at stationary phase, and total concentration of EDTA and sodium metasilicate in the media, which was assumed to be 0.02% w/w in cultures containing 0.1% v/v K30.

The CCA plots in Figures 4C,D, which compare results from 0 and 0.1% v/v K30, include the total array of detected metabolites (text), listed quantitative descriptors (lines), and transformed data after dimension reduction (glyphs). For the 2P08AA and 50v1 strains, stronger correlations along CCA Axis 1 (67.18, 82.53%) are observed when compared to CCA Axis 2 (32.81, 11.10%), respectively. Dimension reduction for the metabolomes from the 2P08AA and 50v1 strains (Figures 4C,D) respectively show clear separations between samples prepared in the absence and presence of 0.1% v/v K30. These trends suggest that the extracellular metabolomic compositions of both strains are impacted by K30 during cultivation. Described in the following sub-sections are comparisons to the quantitative descriptors. For selected metabolites, additional explanations and/or fatty acid abbreviations are provided in parentheses.

#### 3.6.2. Comparisons to survival and growth rates

When considering survival and cultivations, the vectors for plate counts and growth rates for the 2P08AA strain (Figure 4C) overlap with the reduced terms associated with 0% v/v K30 (red diamonds). These correlations suggest that survival and growth rate trends for the 2P08AA strain (across 0 to 0.1% v/v K30) are associated with minimal changes in the extracellular metabolome. These assessments are roughly consistent with the associated univariate analyses that indicate quantitative survivals, ~18% decreases in growth rate, and a lack of changes in the extracellular metabolome (after correction for multiple testing).

From the CCA plot, metabolites from the 2P08AA strain that trend alongside the plate counts *and* growth rates include 2-isopropylmalic acid, pyrophosphate, phosphate, arachidic acid (fatty acid; 20:0), lauric acid (fatty acid; 12:0), capric acid (fatty acid; 10:0), 4-hydroxyphenyllactic acid, sorbitol, and isopentadecanoic acid (13-methyl-14:0). These trends for the 2P08AA strain suggest that survival (which was not impacted) and growth rates (which minimally decreased) relate to a lack of change in the abundances of extracellular saturated fatty acids, phosphate, pyrophosphate, and α-hydroxy acids (2-isopropylmalic acid and 4-hydroxyphenyllactic acid). However, the potential *increases* in extracellular sorbitol (~5-fold increase, *p <* 0.05; Table 1) are suggestive of efflux of a compatible solute.

For the 50v1 strain (Figure 4D), in contrast, the vector describing plate counts overlaps with the reduced terms associated with 0% v/v K30 (pink diamonds), while the vector describing growth rates moderately overlaps with the reduced terms associated with 0.1% v/v K30 (black triangles). These opposing correlations suggest that survival trends (across 0 to 0.1% v/v K30) are associated with minimal changes in the extracellular metabolome, while growth rate trends (across 0 to 0.1% v/v K30) are associated with more notable changes in the extracellular metabolome.

Metabolites trending alongside the plate counts for the 50v1 strain include 2-isopropylmalic acid, 2-hydroxyglutarate, succinic acid, and α-ketoglutarate. These results suggest that survival for the 50v1 strain is related to changes in extracellular α-hydroxy acids (2-isopropylmalic acid, 2-hydroxyglutarate, and α-ketoglutarate) and a dicarboxylic acid (succinate); while univariate tests support ~9-fold decreases in extracellular α-ketoglutarate. Metabolites trending alongside the growth rates for the 50v1 strain include tagatose and N-acetylglucosamine, which suggests that changes in extracellular monosaccharides relate to maintaining stable growth rates.

#### 3.6.3. Comparisons to maximum relative biomass and chelator concentrations

When considering changes in the maximum relative biomass (stationary phase) and chelator concentrations (EDTA and sodium metasilicate from the K30 formulation), similar trends are observed for the 2P08AA and 50v1 strains (Figures 4C,D). The vectors describing chelator concentrations for both strains overlap with the reduced terms associated with 0.1% v/v K30 (black triangles). These correlations suggest the extracellular metabolomes from both strains adjust in response to the change in chelator concentration across 0 to 0.1% v/v K30 (e.g., the metal sequestering agents of EDTA and sodium metasilicate).

From the CCA plot for the 2P08AA strain, metabolites trending alongside changes in the maximum relative biomass and/or chelator concentration include threonate, fructose, oxalate, galactonate, gluconate, gluconolactone, mannose, galactose, and glycerate. These trends suggest that chelator concentrations influence the abundances of extracellular monosaccharides and sugar acids. While these trends are consistent with univariate tests (*p* < 0.05), no support for changes in the abundances of the associated metabolites (Table 2) are obtained after correction for multiple testing (FDR ≤ 0.20).

For the 50v1 strain, metabolites trending alongside changes in the maximum relative biomass and/or chelator concentrations include N-acetylglucosamine, tartrate, 2-methylglycerate, gluconolactone, glycerate, mannose galactonate, and gluconate. These results suggest that the chelator concentrations influence the abundances of extracellular monosaccharides, modified monosaccharides, and sugar acids. These assessments generally are consistent with univariate analyses.

## 4. Discussion

### 4.1. Spacecraft-associated *Acinetobacter*

Molecular insights into the tolerance of spacecraft-associated *Acinetobacter* toward Kleenol 30, a NASA cleanroom floor detergent, were obtained through cultivation, kinetic, and metabolomic approaches. Comparisons were conducted using *A. johnsonii* 2P08AA and *A. radioresistens* 50v1, which were isolated at, respectively, unique locations within the spacecraft assembly facilities, which were subjected to differing cleaning regimes.

The strain *A. johnsonii* 2P08AA was isolated from the floor of the Mars Phoenix lander assembly facility, which was routinely cleansed with Kleenol 30. The strain *A. radioresistens* 50v1 was isolated from the surface of the pre-flight Mars Odyssey orbiter, which was likely cleaned with isopropyl alcohol (2-propanol, isopropanol, IPA) and never exposed to Kleenol 30.

Hence, comparisons across these differing sub-environments provide initial insights into the impacts of repeated cleansing, under the conditions of spacecraft assembly, toward biochemical and cultivation selection.

### 4.2. Survival of the spacecraft-associated *Acinetobacter* against Kleenol 30

Cultivations containing 0–2.0% v/v Kleenol 30 (K30) were conducted in a low-osmolarity minimal media (0.2x M9, 25 μM Fe) containing 150 mM ethanol, which served as the sole supplied carbon source. Cultivation media containing 2.0% v/v K30 were bactericidal for the tested *Acinetobacter*, which suggests a biocide-type of activity for K30.

In contrast, in 1.0% v/v K30, appreciable cultivation tolerances were observed for the 2P08AA and 50v1 strains. Cultures containing 1.0% v/v K30 exhibited (1) ~20–30% survivals, (2) ~66 and 12% decreases in growth rates, and (3) eliminated lag phase times for the 2P08AA and 50v1 strains, respectively. These cultivation tolerances of <2% v/v K30 are significant since current NASA planetary protection practices utilize ~0.8-1.6% v/v K30 for floor cleansing. Our results, therefore, suggest that the spacecraft-associated *Acinetobacter* harbor a tolerance toward the current floor cleansing practices for NASA cleanrooms.

In 0.1% v/v K30, differential impacts were observed across the cultivation parameters. For the 2P08AA strain, cultures exhibited (1) no measurable changes in cell density at late-log phase (plate counts), (2) modest decreases in the growth rate (~18%), and (3) negligible times in the lag phase. These results indicate that K30 impacts the 2P08AA strain by accelerating growth out of the lag phase, slightly inhibiting growth during the exponential phase, and by yielding no changes cell density after cultivation to late-log phase.

For the 50v1 strain, cultures exhibited (1) ~50% survivals, (2) no changes in growth rate, and (3) ~70% decreases in the lag phase time. This is indicative of K30 impacting the 50v1 strain by mildly accelerating growth out of the lag phase, yielding no change in the growth rates during the exponential phase, and inhibiting cell density by ~50% when cultivated to late-log phase.

The differential cultivation trends observed in 0.1% v/v K30 are indicative of strain and growth-phase dependent responses to K30. In turn, these trends suggest that K30 reduces nutrient bioavailability in culture (e.g., alkaline earth and transition metals) and/or elicits specific biochemical responses (e.g., metabolism of K30 and/or enzymatic inhibition by K30) – which together manifest as acceleration out of the lag phase for both strains, slight inhibition in rates of cell division during the exponential phase for the 2P08AA strain, and moderate reductions in total cell density at late-log phase for the 50v1 strain.

### 4.3. Metabolomic profiling of the cultivation tolerances against Kleenol 30

To obtain metabolic insights into the tolerances toward Kleenol 30 (K30), we used cultures grown in 0.1% v/v K30 to obtain reliable biomasses at late-log phase for both strains (see comparison of 0.1 and 1.0% w/w K30 in Figures 1D). For *A. johnsonii* 2P08AA, cultivations with 0.1% v/v K30 exhibited (1) no significant and discreet changes in the abundances of intracellular metabolites, (2) several significant extracellular changes when considering broader classifications such as sugar acids, monosaccharides, organic acids (di- and monocarboxylic acids), and fatty acids, and (3) potential support for biodegradation and metabolism of both detergents in the K30 formulation.

The combined results for the 2P08AA strain suggest that the native and intracellular metabolic state of the 2P08AA strain readily accommodates cultivation in the presence of 0.1% v/v K30. The trends for the 2P08AA strain also suggest that survival is associated with adjustments in the extracellular metabolome and a potential for biodegradation of the Kleenol 30 detergents.

For *A. radioresistens* 50v1, cultivations in 0.1% v/v K30 exhibited (1) differential changes in the abundances of intracellular amino acids, compatible solutes, nucleotide-related metabolites, dicarboxylic acids, and saturated fatty acids, (2) substantial and differential changes in the abundances of extracellular sugar acids, monosaccharides, and organic acids, and (3) *no* clear statistical support for biodegradation and metabolism of the detergents from the K30 formulation (when excluding the potential for degradation *via* benzene metabolism).

These results for the 50v1 strain indicate that the intracellular and extracellular metabolomes of the 50v1 strain significantly adjust to accommodate cultivation in the presence of 0.1% v/v K30. These trends also suggest that survival is associated with intracellular changes to amino acid and peptide metabolism, nucleotide metabolism, central metabolism, fatty acid metabolism, and pyrimidine metabolism.

For the 50v1 strain, the changes in amino acid metabolism include decreases in lysine, proline, glycine, ornithine, homoserine, valine and others listed in Table 1. This is relevant since decreases in lysine and valine are observed during biofilm formation in *A. baumanni* 1,656–2 (Yeom et al., 2013), while decreases in amino acid metabolism are observed after treatments of *A. baumanni* AB5075 with mixed antibiotics (polymyxin B and rifampicin) (Zhao et al., 2021). In *E.coli* UTI189, decreases in intracellular lysine and valine are associated with biofilm formation (Lu et al., 2019). In *Bifodobacterium bifudum*, decreases in intracellular lysine, arginine, and proline are associated with biofilm formation (Sadiq et al., 2020). These observations suggest that the *Acinetobacter* undergo stress-like responses toward K30 that inhibit total cell mass (e.g., survival), which could include changes in early biofilm development.

Trends across nucleotide abundances and the cultivation terms are similarly suggestive of early biofilm-type development (Sarkar and Chakraborty, 2008; Castillo-Juarez et al., 2017) and/or quorum sensing related changes such as intracellular PHA synthesis (Kessler and Witholt, 2001; Xu et al., 2011). These trends include increases in the maximum relative biomass terms for the 2P08AA and 50v1 strains, decreases in 5′-MTA and 5’-AMP for the 50v1 strain, and substantial increases in extracellular monosaccharides (e.g., mannose, galactose, and fructose for the 2P08AA strain).

In comparison, biofilms of *E. coli* UTI189 show decreases in 5′-MTA and 5’-CMP, as well increases in metal-binding siderophores, when Fe^3+^ concentrations in culture decrease from 10 to 1 μM (Guo et al., 2021). Lowered 5′-MTA abundances are associated with biofilms of *Pseudomonas aeruginosa* PAO1 (Tielen et al., 2013). In *Pseudomonas fluorescens* PF08, exogenously added 5’-AMP inhibits biofilm formation (Wang et al., 2021). In *Streptococcus pyogenes*, the import of mannose induces lysozyme resistance *via* a quorum sensing pathway (Chang et al., 2015); while exogenously added galactose differentially inhibits and activates biofilm development in differing species of *Streptococcus* (Ryu et al., 2016).

### 4.4. Biochemical strategies against Kleenol 30

To obtain biochemical insights into the mechanisms of tolerance, the metabolomic trends were interpreted in context of the cultivation conditions. In this study, cultivations were conducted in low-osmolarity media (0.2x M9) supplemented with 25 μM Fe^2+^ (Fe(NH_4_)_2_(SO_4_)_2_), which slowly oxidized to Fe^3+^ under the aerobic cultivations in the capped glass tubes (per absorbance spectroscopy). However, in control experiments, cultures supplemented with [Fe(EDTA)]^1−^ grew slower, when compared to Fe(NH_4_)_2_(SO_4_)_2_, while cultivations in acid-washed glassware yielded no growth. Therefore, under the cultivation conditions, trace elements and species such as Zn^2+^, Co^2+^, Cu^2+^, Mn^2+^, BO_3_^3−^ (borate; a B source), and MoO_4_^2−^ (molybdate; a Mo source) were likely obtained from trace contaminants in the media and/or the glassware.

These details are relevant since K30 contains EDTA and sodium metasilicate, which are chelators that sequester transition and alkaline earth metals. Hence, in culture, chelation by EDTA would likely restrict the bioavailability of transition metals and inhibit growth – as observed in control studies. Limited bioavailabilities for Ca^2+^ and Mg^2+^ are also expected since sodium metasilicate (Na_2_SiO_3_) is a water softening agent that functions through ion exchange *via* chelation of Ca^2+^ or Mg^2+^ (and release of Na^+^).

Under these considerations, therefore, our review of the extracellular metabolomes reveals changes in several biochemicals possessing metal-binding properties. Appraisal of the literature shows that sugar acids such as galactonate, gluconate, and gluconolactone form appreciably stable complexes with transition metals such as Fe^3+^, Zn^2+^, Co^2+^, Cu^2+^, and Mn^2+^ (Sawyer, 1964; Carper et al., 1989; Frutos et al., 1998; Gyurcsik and Nagy, 2000). For the alkaline earth metals of Ca^2+^ and Mg^2+^, stable complexes are formed with sugar acids such as gluconolactone and glycerate (Taga et al., 1978; Meehan et al., 1979; Tajmir-Riahi, 1990), monosaccharides such as mannose (Angyal, 1973), and organic acids such as oxalate (Chutipongtanate et al., 2013; Izatulina et al., 2018) and tartrate (Hawthorne et al., 1982). In addition, borate and Ca^2+^ form stable complexes with gluconate, glycerate, and tartrate (van Duin et al., 1987); while molybdate yields stable complexes with mannose and fructose (Spence and Kiang, 1963; Sauvage et al., 1996).

Biochemically, therefore, these observations suggest that the 2P08AA strain targets trace Ca^2+^ and Mg^2+^ acquisition through potential increases in extracellular glycerate, gluconolactone, and oxalate, but not *via* gluconate and galactonate, which bind transition and alkaline earth metals but were not detected. In contrast, the 50v1 strain potentially targets both transition and alkaline earth metals given the increases (*p <* 0.05, FDR ≤ 0.20) in extracellular gluconate, galactonate, glycerate, gluconolactone, and tartrate. In fact, increases in glycerate export for the 50v1 strain are supported by the increases in extracellular glycerate and decreases in intracellular glycerate. Control of intracellular Ca^2+^ retention is also suggested by increases in extra- and intracellular tartrate. Multivariate tests support these total trends and suggest that chelator concentrations (from the K30 formulation) influence the abundances of extracellular metal-binding biochemicals.

Thus, our total results suggest that up-regulations (*per se*) in extracellular trace element acquisition strategies are common survival features to the spacecraft-associated *Acinetobacter*. Future studies using concentrated and clarified cultivation media may yield spectral support for the trace metal chelation. Additional common survival features may include regulation of the bulk osmolarity, as suggested by decreases in intracellular compatible solutes for the 50v1 strain (e.g., glycerate, 3-phosphoglycerate, and mannitol), statistical associations (CCA plot) between survival and changes in intracellular compatible solutes (e.g., trehalose) for the 2P08AA strain, and increase in an extracellular compatible solute for the 2P08AA strain (e.g., sorbitol).

When considering intracellular changes, differing responses to 0.1% v/v K30 are observed by the 2P08AA and 50v1 strains. While limited intracellular responses are observed for the 2P08AA strain, substantial intracellular changes are observed for the 50v1 strain. Multivariate tests for the 50v1 strain support a potential for the synthesis of light scattering PHAs during stationary phase in response to 0.1% v/v K30. Relatedly, univariate tests suggest decreases in the export of α-ketoglutarate and 4-hydroxybutanoate (e.g., decreases in extracellular abundances), while multivariate tests show correlations between intracellular PHA synthesis (e.g., α-ketoglutarate, 4-aminobutanoate, and 4-hydroxybutanoate) and increases in the *apparent* maximum relative biomass.

For the 2P08AA strain, multivariate analyses highlight a potential for partial metabolism of the detergents (from the Kleenol 30 formulation) – which would serve as an additional biochemical strategy toward survival. While these exact trends were not observed for the 50v1 strain, multivariate tests do support correlations between survival and changes in the degradation of aromatic compounds (benzene metabolism). In support, GC–MS studies in Mogul et al. (2018) provided evidence for scission of polyethylene glycol mono-nonylphenyl ether by the 50v1 strain. Future targeted cultivation studies are required to confirm the extent of biodegradation of the detergent by these strains.

When considering impacts of K30 on the lag phase, we observe a differential concentration dependence, where the time in lag phase is eliminated in the presence of 0.1 v/v K30 for the 2P08AA strain, and in 1.0% v/v K30 for the 50v1 strain. We venture that metabolism of the alkyl sides chains on the detergents (*via* ω-oxidation of the terminal carbon and subsequent cycles of β-oxidation) may serve as activators for acceleration out of the lag phase, and that degradation of the aromatic detergent cores (*via* benzene metabolism) may sustain the 50v1 strain during exponential phase, where growth rates show no inhibition in the presence of 0.1% v/v K30. If so, carbon and energy acquisition from the detergents could serve as an added biochemical strategy toward survival. Alternatively, the K30 detergents could potentially disperse any low abundance cellular aggregates during the lag phase, thereby yielding higher OD values and the apparent increases in biomass. Under this assumption, the observed concentration dependence across the strains would be suggestive of the cellular aggregates from both strains having differing stabilities.

## 5. Conclusion

In conclusion, our combined cultivation and metabolomic results lend supports toward the hypothesis that repetitive cleansing with Kleenol 30 presents as selective pressures toward members of the spacecraft microbiome – where the phenotypic outcomes likely include cultivation tolerances and measurable biochemical responses. Fittingly, our described results indicate that Kleenol 30 is more readily tolerated by the floor-associated *A. johnsonii* 2P08AA, when compared to the spacecraft surface-associated *A. radioresistens* 50v1. However, both strains display reasonable tolerances toward 1% v/v Kleenol 30, which is higher than the current formulation (~0.8-1.6% v/v) used in NASA planetary protection practices. These results indicate that the spacecraft-associated *Acinetobacter* may harbor tolerances toward the floor cleaning conditions.

Such tolerances are likely important considerations for lower bioburden planetary missions, such as orbiter missions to Europa (Planetary Protection Category III), life detection missions to Mars (Planetary Protection Category IVb) or Europa (Planetary Protection Category IV), and investigations of Mars Special Regions (Planetary Protection Category IVc). In this context, our results suggest that higher floor cleanliness levels may be achieved with higher concentrations of Kleenol 30 (e.g., 2–3% v/v) or rotations with differing concentrations of Kleenol 30 (e.g., 1 and 3% v/v). Alternatively, rotation of differing floor cleansers could assist in achieving lower floor bioburdens (e.g., Kleenol 30 and a quaternary ammonium compound), with the caveat that the detergents and/or biocides retain compatibility with the spacecraft assembly procedures and spacecraft materials.

## Data availability statement

Metabolomic data (Study ID ST002380, DatatrackID: 3552) are available through the Metabolomics Workbench (https://www.metabolomicsworkbench.org/) at the NIH Common Fund’s National Metabolomics Data Repository (NMDR) and can be accessed directly via the project DOI: http://dx.doi.org/10.21228/M8XH73.

## Author contributions

RM, DM, SL, and BR contributed to analysis of the data, assisted in drafting of the report, approved of submission of the manuscript. RM analyzed the metabolomic data, performed the statistical analyses, prepared the manuscript, and is the corresponding author. DM and BR conducted the survival assays and cultivation kinetics. SL prepared cultures for metabolomics analysis. All authors contributed to the article and approved the submitted version.

## Funding

This work was supported by a grant (NNH18ZDA001N) from the Planetary Protection Research component of the National Aeronautics and Space Administration (NASA) Research Opportunities in Space and Earth Sciences program.

## Conflict of interest

The authors declare that the research was conducted in the absence of any commercial or financial relationships that could be construed as a potential conflict of interest.

## Publisher’s note

All claims expressed in this article are solely those of the authors and do not necessarily represent those of their affiliated organizations, or those of the publisher, the editors and the reviewers. Any product that may be evaluated in this article, or claim that may be made by its manufacturer, is not guaranteed or endorsed by the publisher.

## Footnotes

^1^ http://metamapp.fiehnlab.ucdavis.edu/ocpu/library/MetaMapp2020/www/

^2^ https://cytoscape.org/

^3^ https://chemrich.fiehnlab.ucdavis.edu/
